# Pyk2 regulates sepsis-induced lung injury via ferroptosis

**DOI:** 10.22038/IJBMS.2023.69578.15153

**Published:** 2023

**Authors:** Jia Wang, Pengtao Bao, Yugeng Liu

**Affiliations:** 1 Beijing Key Laboratory of Cardiopulmonary Cerebral Resuscitation, Capital Medical University, Beijing, People’s Republic of China; 2 Emergency Medicine Clinical Research Center, Beijing Chao-Yang Hospital, Capital Medical University, Beijing, People’s Republic of China; 3 Clinical Center for Medicine in Acute Infection,Capital Medical University, Beijing, People’s Republic of China; 4 The Eighth Medical Center, PLA General Hospital, Beijing, People’s Republic of China; # These authors contributed equally to this work

**Keywords:** Ferroptosis, Inflammatory mediators, Macrophage activation, Protein tyrosine kinase, Sepsis

## Abstract

**Objective(s):**

The onset of sepsis represents a hyper-inflammatory condition that can lead to organ failure and mortality. Recent findings suggest a potential beneficial effect of protein tyrosine kinase Pyk2 inhibitor on sepsis in a mouse model. In this study, we investigated the regulatory role of Pyk2 inhibitor in ferroptosis and sepsis-associated acute lung injury (ALI).

**Materials and Methods:**

A Pyk2 inhibitor or a ferroptosis regulator were injected into mice sustaining sepsis-induced ALI and the effects on lung injury and pro-inflammatory response were evaluated. Clinically, Pyk2 expression was determined in serum samples of patients with sepsis. Further, the association between serum Pyk2 levels and clinical features was determined.

**Results:**

Experimental mouse models revealed that treatment with Pyk2 inhibitor TAE226 can significantly alleviate lung injury, downregulate pro-inflammatory responses and decrease markers of ferroptosis, which were induced by LPS. Both upregulation and downregulation of ferroptosis can lead to the loss of TAE226 function, indicating that Pyk2 promotes inflammation via ferroptosis induction. Analysis of clinical samples revealed that the serum Pyk2 levels were significantly increased in patients with sepsis. The serum Pyk2 levels were associated with APACHE2 scores and 30-day mortality. Further, we found a negative correlation between serum Pyk2 and Fe^3+^ levels, which was consistent with the mechanism identified in the mouse model.

**Conclusion:**

Pyk2 inhibitor of ferroptosis is a promising therapeutic candidate against sepsis-related ALI.

## Introduction

Sepsis is a life-threatening organ dysfunction caused by dysregulated host response to infection, which can lead to severe sepsis or septic shock. The patients often develop secondary acute organ dysfunction, severe sepsis, and hypotension that cannot be reversed by fluid resuscitation (1). The lungs are particularly susceptible to injury during sepsis, and the primary risk factors for acute lung injury (ALI) in > 50% patients were attributed to sepsis (2). As the most common immune cells in lungs under homeostasis, macrophages play a crucial role in sepsis-induced ALI (3). The macrophages are recruited and activated by lipopolysaccharide (LPS) and the originally resident alveolar macrophages can release pro-inflammatory cytokines, which are the main factor contributing to lung injury (4).

 Macrophages have been implicated in pathological cell death associated with several health conditions, including degenerative diseases (5), carcinogenesis (6), and ischemia-reperfusion injury (7). Recent evidence indicates that cell death induces pro-inflammatory (M1) and anti-inflammatory (M2) polarization of macrophages, which play an important role in lung injury (8). There are many types of programmed and non-programmed cell death, including apoptosis, autophagic death, necrosis, pyroptosis and ferroptosis (9-11). The signaling pathways in these cell deaths differ, and usually exhibit cross-talk (10-11). Our group previously reported that ferritin/Fe^3+^, which is strongly associated with ferroptosis (12), is an independent risk factor affecting the prognosis of patients with sepsis (1), indicating that ferroptosis might be involved in sepsis and sepsis-induced ALI. 

FAK and Pyk2 are members of the FAK family, which plays a critical role in modulating the cytoskeleton and other cellular structures to regulate macrophage polarization (13). Pharmacological inhibition of FAK-Pyk2 pathway protects various organs, including lung, liver and spleen, against inflammatory damage in septic mice (14-15). However, the regulatory mechanisms are still poorly understood.

Pyk2 has been implicated in many types of cell death (14), but its association with ferroptosis has yet to be reported. In this study, we explored the regulatory role of Pyk2 inhibitor in ferroptosis and sepsis-associated ALI. 

## Materials and Methods

***Ethics approval and consent to participate***

This study was approved by the ethics committee of Beijing Chaoyang Hospital. The informed consent of patients or family members was obtained, and patient confidentiality was strictly maintained. All patients provided written informed consent and compliance with the Declaration of Helsinki. The animal experimental protocol was approved by the Institutional Animal Care and Use Committee of Beijing Chaoyang Hospital.

***Clinical samples***

We collected samples from 482 patients diagnosed with sepsis in the emergency department and Intensive Care Unit (ICU) wards of Beijing Chao-Yang Hospital from January 2012 to June 2020, and their sera were maintained in the specimen bank of our laboratory. The detailed inclusion and exclusion criteria were described previously (1). Briefly, sepsis was diagnosed according to the 2016 diagnostic criteria for sepsis and septic shock published in the Journal of the American Medical Association, sepsis 3.0. The inclusion criteria were: 1) age above 18 years; 2) samples collected within 24 hr after the onset of sepsis following admission; and 3) patients without exposure to medication before data collection. The exclusion criteria were: 1)  diseases that increase or decrease transferrin levels, such as acute viral hepatitis, rheumatoid arthritis, primary renal disease, systemic lupus erythematosus, history of hemodialysis, and a combination of malignant tumors; 2) drug treatment prior to data collection; 3) patients with incomplete clinical data or those who were hospitalized for the second time; and 4) patients who voluntarily dropped out of the study during treatment. In this study, we calculated the sample size using PASS 15.0 software. The “Test for One ROC Curve” was used under the following parameters: power=0.8; alpha=0.05; group allocation: Equal (N+=N-); AUC 0=0.6; AUC1=0.8; lower FPR= 0.00; upper FPR=1.00; type of data: Discrete (Rating); B (SD Ratio=SD-/SD+)=1.0. Based on the procedure and parameters above, the calculated minimum sample size (n) was 58. Therefore, the sera from 58 patients with sepsis were randomly selected from the specimen bank. A group of 58 age- and gender-matched healthy volunteers were included as healthy controls (HCs) and their sera were collected. 

***Animal model of sepsis-induced ALI***

Six- to eight-week-old C57BL/6J mice, including a random selection of females and males, were randomly allotted to different groups, respectively. The mice were pre- or post-treated with TAE226 at doses of 10 mg/kg, 20 mg/kg, 40 mg/kg or saline for 2 hr. Endotoxic shock was induced via intraperitoneal injection of LPS at a dose of 20 mg/kg or 40 mg/kg. Saline was used as the control. Serum samples were collected after 6 hr and lungs were collected after 12 hr. The inflammatory injury to lung tissue was determined using Hematoxylin & Eosin stain and specific indicators were determined as reported previously (2). The mice were injected with LPS intraperitoneally, and their survival was monitored every hour.

***Measurement of cytokines, iron and GSH levels***

Cytokines, including TNF-α, IL-1β, and IL-6, were quantified via ELISA (Jiangsu Meimian industrial Co., Ltd, Yancheng, China), according to the manufacturer’s instructions. The tissue iron concentrations were also determined with ELISA as instructed. Total GSH and oxidized GSH concentrations in cells and tissue were determined via T-GSH/GSSG Detection Assay, according to the manufacturer’s protocol.

***Reverse transcription-quantitative polymerase chain reaction (qRT-PCR)***

We used TRIzol reagent (Invitrogen, Grand Island, NY, USA) to obtain the total RNA from the samples. Subsequently, reverse transcription-quantitative polymerase chain reaction (qRT-PCR) assay was performed using the PrimeScript RT-PCR kit (Takara, Bio, Inc., Shiga, Japan). The IQ5 fluorescence quantitative PCR detector (Bio-Rad, Hercules, CA, USA) was used to perform qRT-PCR. The primer sequences of the targets are as follows (5’ -> 3’):

PTGS2:

Forward Primer, TTCCAATCCATGTCAAAACCGT;

Reverse Primer, AGTCCGGGTACAGTCACACTT;

SLC7A11:

Forward Primer, GGCACCGTCATCGGATCAG;

Reverse Primer, CTCCACAGGCAGACCAGAAAA;

GPX4:

Forward Primer, TGTGCATCCCGCGATGATT;

Reverse Primer, CCCTGTACTTATCCAGGCAGA;

ACSL4:

Forward Primer, CCTGAGGGGCTTGAAATTCAC;

Reverse Primer, GTTGGTCTACTTGGAGGAACG;

***Western blotting analysis***

Protein extracts were prepared in protein lysis buffer (Pierce, Thermo Fisher Scientific). The protein was separated by SDS-PAGE and transferred to PVDF membrane. After blocking, the membrane was incubated with the primary antibody overnight at 4 ^º^C, followed by incubation with the secondary antibody. Protein bands were observed by enhanced chemiluminescence and film exposure.

***Statistical analysis***

Data are expressed as the mean±standard deviation from≥3 separate experiments performed in triplicate. For continuous variables, the differences between two groups were determined using the two-tailed Student’s t-test. The differences between more than two groups were determined via ANOVA. For discrete variables, the differences between two groups were compared using Pearson’s Chi-square test. The Kaplan-Meier method was used to estimate overall survival and the survival rates were determined using the Log-rank test. All the statistical analyses were performed with GraphPad prism 8. 

**Figure 1:**
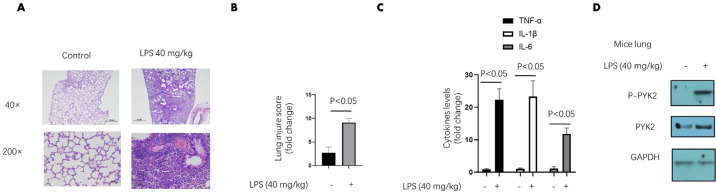
Pyk2 level and phosphorylation are up-regulated in sepsis induced lung injure mouse model

Mice were treated with LPS (40 mg/kg) for 12 hr. Subsequently, the lung tissues were harvested. (A) The histopathology of the lungs from the mice was determined by H&E stain. (B) Lung injure scores were determined. (C) Cytokines, such as TNF-α, IL-1β, and IL-6 in the lungs from the mice were determined by ELISA. (D) Pyk2 level and phosphorylation were determined by western blotting assay. Each treatment group was composed of six mice

**Figure 2:**
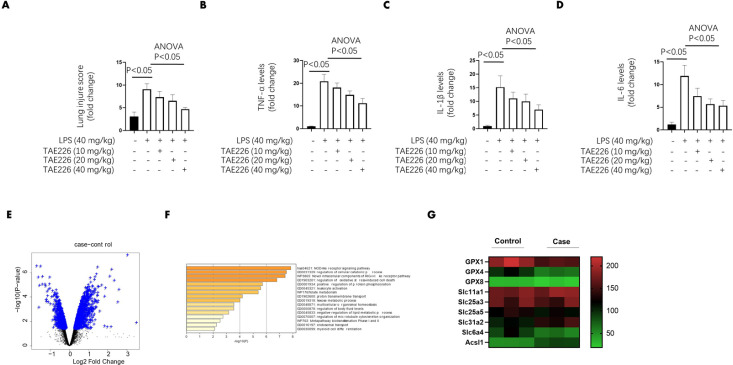
Pyk2 activation play a key role in sepsis induced mice lung injury

Mice were pretreated with or without multiple doses of TAE226 (10 mg/kg and 40 mg/kg), a spesific Pyk2 activation inhibitor for 2 hr, followed by LPS challengement (40 mg/kg) for 12 hr. (A) Lung injure scores were determined. (B-D) Cytokines, such as (B) TNF-α, (C) IL-1β, and (D) IL-6 in the lungs from the mice were determined by ELISA. (E-G) The lung tissues in LPS treated group (control group) and LPS plus high dose TAE226 (40 mg/kg) treated group (case group) were harvested and were used to determine the RNA-seq assay. (E) Volcano plot displays the number of genes with significant differences between the case and control group with the cutoff value at 2. (F) KEGG pathway enrichment for the identified differential targets described in (E). (G) Heatmap analysis displaying the ferroptosis related targets described in (E). Each treatment group was composed of six mice

**Figure 3:**
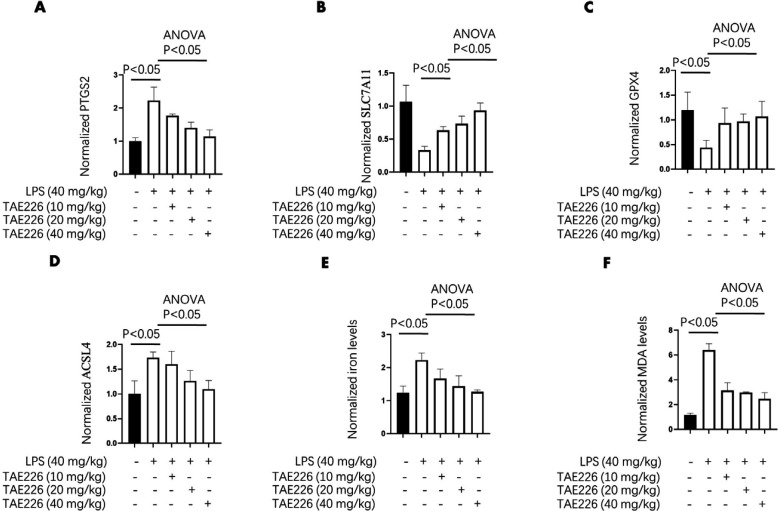
Pyk2 inhibitor decreased ferroptosis associated markers in LPS-induced sepsis mouse model

Mice were pretreated with high (40 mg/kg) or low doses (10 mg/kg) of TAE226, followed by LPS challengement (40 mg/kg) for 12 hr Subsequently, the lung tissues were harvested and were used to performe the qRT-PCR assay on (A) PTGS2; (B) SLC7A11; (C) GPX4 and (D) ACSL4 levels. Meanwhile, the lung tissues were harvested and were used to determine (E) iron and (F) MDA levels. Each treatment group was composed of six mice

**Figure 4:**
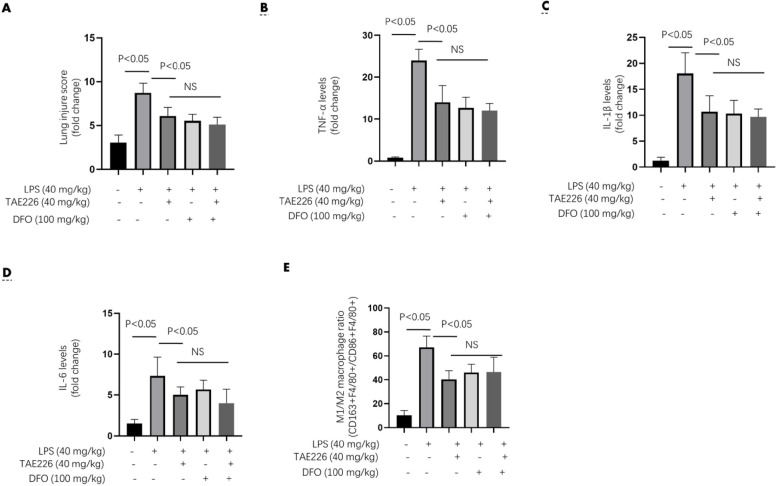
Pyk2 inhibitor alleviated ALI damages and pro-inflammatory responses similar as iron chelator in LPS-induced sepsis mouse model

Mice were pretreated with TAE226 at 40 mg/kg (TAE226 group), iron chelator deferoxamine (DFO group) at 100 mg/kg, or TAE226 (40 mg/kg) plus DFO (100 mg/kg) (TD-comb group) for 2 hours, followed by LPS challengement (40 mg/kg) for another 12 hours. (A) Lung injure scores were determined. (B-D) Cytokines, such as (B) TNF-α, (C)IL-1β, and (D)IL-6 in the lungs from the mice were determined by ELISA. (E) M1/M2 macrophage ratio in in the lungs from the mice were determined by flow-cytometry. Each treatment group was composed of six mice

**Figure 5:**
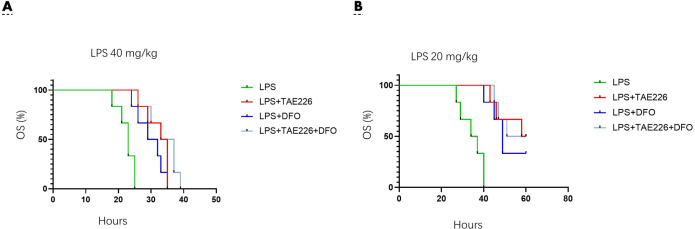
Pyk2 inhibitor results in reduced mortality of mice challenged with lipopolysaccharide (LPS)

Mice were pretreated with TAE226 at 40 mg/kg (TAE226 group), iron chelator deferoxamine (DFO group) at 100 mg/kg, or TAE226 (40 mg/kg) plus DFO (100 mg/kg) (TD-comb group) for 2 hr, followed by LPS challengement at (A) 40 mg/kg or (B) 20 mg/kg, and mortality was observed. The Kaplan–Meier method was used to estimate overall survival and survival rates were determined using the Log-rank test. Each treatment group was composed of six mice

**Figure 6:**
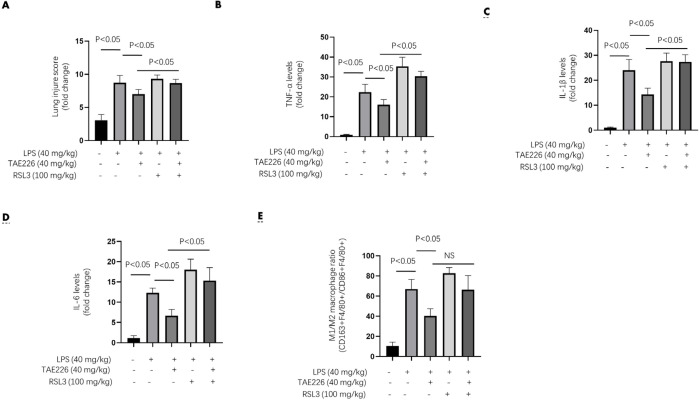
GPX4 inhibitor reverses the function of Pyk2 inhibitor in ALI damages and pro-inflammatory responses

Mice were treated with or without TAE226 at 40 mg/kg (TAE226 group), GPX4 inhibitor RSL3 (RSL3 group) at 100 mg/kg or TAE226 (40 mg/kg) plus RSL3 (100 mg/kg) (TR-comb group) for 2 hours, followed by LPS challengement (40 mg/kg) for another 12 hr. (A) Lung injure scores were determined. (B-D) Cytokines, such as (B) TNF-α, (C)IL-1β, and (D)IL-6 in the lungs from the mice were determined by ELISA. (E) M1/M2 macrophage ratio in in the lungs from the mice were determined by flow-cytometry. Each treatment group was composed of six mice

**Figure 7:**
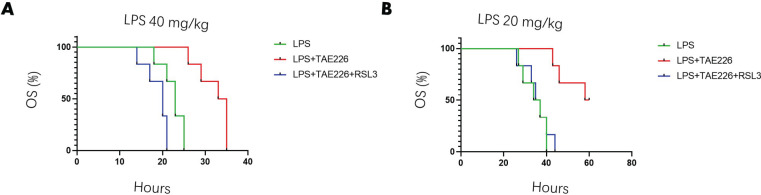
GPX4 inhibitor reverses the function of Pyk2 inhibitor in reducing mortality of mice challenged with lipopolysaccharide (LPS)

Mice were treated with or without TAE226 at 40 mg/kg (TAE226 group), GPX4 inhibitor RSL3 (RSL3 group) at 100 mg/kg or TAE226 (40 mg/kg) plus RSL3 (100 mg/kg) (TR-comb group) for 2 hr, followed by LPS challengement at (A) 40 mg/kg or (B) 20 mg/kg, and mortality was observed. The Kaplan-Meier method was used to estimate overall survival and survival rates were determined using the Log-rank test. Each treatment group was composed of six mice. Each treatment group was composed of six mice

**Figure 8:**
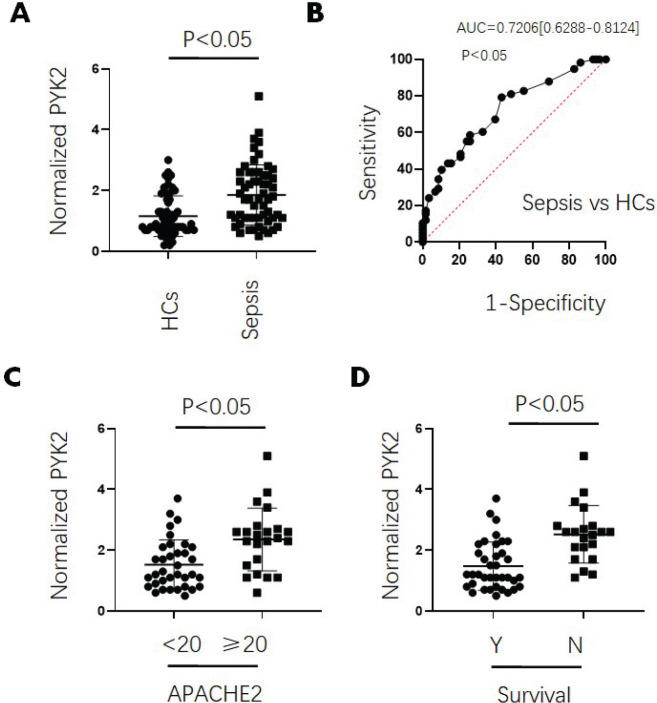
Serum Pyk2 levels in sepsis patients and HCs

(A) Comparison of serum Pyk2 levels between sepsis patients (n=24) and HCs (n=24). (B) correlation between serum Pyk2 levels and sepsis risk by ROC curve. (C) Comparison of serum Pyk2 levels between APACHE2≥20 group (n=8) and APACHE2<20 group (n=16) in sepsis patients. (D) Comparison of serum Pyk2 levels between 30 days death group (n=7) and non-death (n=17) in sepsis patients

**Table 1:** Demographic characteristics of sepsis patients and healthy controls

Detection Index	Healthy controls (n=58)	Sepsis patients (n=58)	P
Age (years)	73.4±14.5	76.1±16.2	0.346
Gender (M/F)	33/25	37/21	0.356
Death (Y/N)	---	18/40	---
APACHE2	11.7±6.6	23.5±8.4	<0.001
Serum Fe^3+^	22.1±11.5	7.7±3.8	<0.001

## Results

***Pyk2 levels and activation were upregulated in a mouse model of sepsis-induced lung injury***

First, mice were challenged with LPS (40 mg/kg) for 12 hr. The lung tissues were harvested to determine the specific indicators. Compared with the control group, the LPS group showed signs of lung injury, including leukocyte accumulation, pulmonary edema and severe pulmonary inflammation (Figure 1A). Lung injury score was determined by analyzing the pathological characteristics, and this score was three-fold higher in the LPS-treated group (Figure 1B). Meanwhile, the proinflammatory cytokines were significantly results indicated successful establishment of a mouse model of sepsis-induced lung injury. We found that the Pyk2 level was slightly upregulated in the LPS-treated group, while its phosphorylation level was increased markedly (Figure 1D). These results prompted us to explore the role of Pyk2 in the pathogenesis of sepsis-induced lung injury.

***Pyk2 activation plays a key role in sepsis-induced lung injury***

Subsequently, mice were pretreated with or without multiple doses of TAE226 (10 mg/kg, 20 mg/kg and 40 mg/kg), a specific Pyk2 inhibitor of activation for 2 hr, followed by LPS challenge (40 mg/kg) for 12 hr. The lung injury score and proinflammatory cytokines induced by LPS were reversed by TAE226 in a dose-dependent manner (Figure 2A-D), indicating that Pyk2 activation played a key role in sepsis-induced lung injury. 

The lung tissues in LPS-treated group (control group) and the group treated with LPS plus high-dose TAE226 (40 mg/kg) (case group) were harvested and used in the RNA-seq assay. With a cutoff line>2-fold and *P≤*0.05, 415 genes were differentially expressed between case and control groups (Figure 2E). GO enrichment analyses revealed that the dysregulated genes were strongly involved in metabolic processes and immune cell activation (Figure 2F). Interestingly, the ferroptosis pathway-related proteins, including GPX4, members of acyl-CoA synthetase long-chain (ASLC) family and the synergistic suppression of the solute carrier (SLC) family were regulated by TAE226 (Figure 2G).

***Pyk2 activation plays a key role in regulating ferroptosis in mice with LPS-induced sepsis***

The regulation of ferroptosis by Pyk2 was investigated by analyzing GPX4, which was identified by RNA-seq. We also tested other important ferroptosis-positive markers, such as ACSL4 and PTGS2 as well as the ferroptosis-negative marker SLC7A11, because their family members were identified by RNA-seq. Data indicated that the levels of PTGS2 and ACSL4 increased, while those of GPX4 and SLC7A11 decreased in lung tissues after LPS treatment; TAE226 pretreatment significantly reversed these effects in a dose-dependent manner (Figure 3A-D). Besides, TAE226 reversed the LPS-induced increase in tissue iron and MDA levels in a dose-dependent manner (Figure 3E-F). These results indicated that the inhibition of Pyk2 activation alleviated ferroptosis-associated markers in a mouse model of LPS-induced sepsis.

***Pyk2 inhibitor alleviated lung injury and pro-inflammatory response similar to iron chelator in mice with LPS-induced sepsis***

To understand the role of ferroptosis in mediating Pyk2 function, we pretreated mice with 40 mg/kg of TAE226 (TAE226 group), 100 mg/kg of iron chelator deferoxamine (DFO group), or TAE226 (40 mg/kg) plus DFO (100 mg/kg) (TD-comb group) for 2 hrs, followed by LPS challenge (40 mg/kg) for another 12 hrs. TAE226 alleviated the LPS-induced lung injury scores and proinflammatory cytokine levels (Figure 4A-D). Considering that these proinflammatory cytokines were mainly secreted by M1 macrophages, we also determined the macrophage M1/M2 ratio by flow cytometry. TAE226 downregulated the LPS-induced M1/M2 ratio (Figure 4E). The DFO treatment strategy, used to inhibit ferroptosis (16), had a similar impact on markers of lung injury. Meanwhile, the above markers of lung injury in TD-comb group were not significantly different from those of the DFO group (Figure 4C-E).

Subsequently, the above groups of mice were used to plot the survival curve (endpoint 60 hr). TAE226 significantly extended survival of mice by several hours after treatment with LPS (<40 mg/kg). The survival curve involving TAE226, DFO and the combination group showed no significant differences between the mice (Figure 5A). Following low-dose LPS challenge (20 mg/kg), pretreatment with TAE226, DFO or TD-comb increased the survival rate at the endpoint of 60 hr (Figure 5B). The differences in survival rate between DFO and TD-comb also did not reach statistical significance (Figure 5A-B). 

Taken together, these data indicated that when ferroptosis was inhibited, Pyk2 lost its regulatory effect in lung injury and pro-inflammatory response in mice with LPS-induced sepsis.

***GPX4 inhibitor reverses the function of Pyk2 inhibitor in LPS-induced sepsis via ferroptosis inhibition***

GPX4 inhibitor RSL3 has been reported as an inducer of ferroptosis (17). Next, we pretreated the mice with 40 mg/kg TAE226 (TAE226 group), 100 mg/kg GPX4 inhibitor RSL3 (RSL3 group) or TAE226 (40 mg/kg) plus RSL3 (100 mg/kg) (TR-comb group) for 2 hr, followed by LPS challenge (40 mg/kg) for another 12 hr. The TAE226 treatment reduced lung injury scores and pro-inflammatory responses, which were reversed by RSL3 (Figure 6A-E). Meanwhile, the decreased survival following TAE226 exposure was also reversed by RSL3 (Figure 7A-B). Taken together, these data indicated that Pyk2 inhibitor alleviated ALI and pro-inflammatory response depending on ferroptosis inhibition.

***Serum Pyk2 levels were increased in patients with sepsis***

We next explored the association between serum Pyk2 levels and clinical features in patients with sepsis. The clinical detection indices of 58 patients with sepsis and 58 HCs are shown in Table 1. There were no significant differences in age or gender between the groups. Similar to our previous report (1), the Fe^3+^ levels were lower in patients with sepsis than in HCs. Serum Pyk2 levels were increased in sepsis (Figure 8A). Meanwhile, the receiver operating characteristic (ROC) curve showed that serum Pyk2 levels were effective in identifying patients with sepsis from HCs with the area under the curve (AUC) at 0.7206 (0.6288-0.8124) (Figure 8B). Further, the patients diagnosed with sepsis in the APACHE2≥20 group and in the group with 30-day death carried higher serum Pyk2 levels than in APACHE2 < 20 group and in 30-day survival group, respectively (Figure 8C-D). These results suggested that Pyk2 was associated with sepsis progression. 

## Discussion

Sepsis is a disease in which pathogenic microorganisms or toxins released by them are disseminated in the body through the blood circulatory system, which triggers inflammatory response and leads to tissue and organ dysfunction (18-19). We still have a long way to go to fully understand sepsis, especially the lack of effective treatment measures to reduce the associated morbidity and mortality. Therefore, early and mid-term differential diagnosis of sepsis is extremely important (20). Our group has been engaged in the study of diagnostic markers of sepsis for a long time, and found that inflammatory markers, such as Fe^3+^, procalcitonin, absolute neutrophil count, and D-dimer, are independent risk factors affecting the prognosis of patients with sepsis (1). Further, we have been identifying new diagnostic markers of sepsis, including Pyk2. In this study, we found that patients with sepsis had markedly high serum Pyk2 levels compared with healthy controls. In addition, high levels of serum Pyk2 were significantly associated with APACHE2 scores and 30-day mortality. 

Pyk2 is a kind of non-receptor protein tyrosine kinase that plays a vital role in various cellular functions, including motility, adhesion, signaling, and gene expression (21). Pyk2 plays a pro-inflammatory role by promoting macrophage M1 polarization, and Pyk2 inhibitors can be used to protect against inflammatory damage in septic mice (14-15). In this study, the Pyk2 inhibitor TAE226 decreased the levels of inflammatory cytokines, alleviated ALI and prolonged the survival time of mice with LPS-induced sepsis, which is consistent with previous reports. Recent studies have shown that the Pyk2 functions may be partly mediated via cell death pathways (17). For example, Lim *et al.* have reported that Pyk2 inhibited P53, and thereby blocked cellular apoptosis (22). Conte *et al.* found that Pyk2 regulated cancer cell autophagy (23). Di Wang *et al.* recently reported that Pyk2 regulated necroptosis in macrophages (24). In this study, we found that ferroptosis, which is a recently recognized cell death mechanism, was associated with Pyk2 function. In the mouse model of sepsis, LPS treatment significantly induced the expression of PTGS2 and SLC7A11, and upregulated the levels of iron and MDA in lung tissues, while Pyk2 inhibitor TAE226 decreased these positive markers of ferroptosis. Further, TAE226 treatment downregulated the negative markers of ferroptosis GPX4 and ACSL4.

As we know, most cell death types, reported to be regulated by Pyk2 previously, exhibit anti-inflammatory effects, which is contrary to the pro-inflammatory role of Pyk2 (17, 24). Ferroptosis is widely confirmed as a cell death mechanism that promotes inflammation. Especially, He *et al.* demonstrated that targeting ferroptosis attenuates the sepsis-induced inflammation and ALI (25). Here, based on multiple lines of evidence, we confirmed that Pyk2 inhibitor TAE226 plays an anti-inflammatory role in a mouse model of LPS-induced sepsis via ferroptosis inhibition. First, the ferroptosis inhibitor DFO alleviated ALI and pro-inflammatory response, which was similar to the role of TAE226. Second, when ferroptosis was inhibited by DFO, TAE226 lost its anti-inflammatory ability. Third, when ferroptosis was induced, the TAE226 function was reversed. Our study reports the existence of a Pyk2-iron-inflammation signal axis mediated by ferroptosis.

Unfortunately, clinical evidence does not suggest that Pyk2 positively regulates ferroptosis, because we found that Fe^3+^ levels were decreased in patients with sepsis carrying high levels of Pyk2. Although some studies have found that high iron levels promote ferroptosis and related inflammation, many clinical data also indicated that iron insufficiency can also induce inflammation (1). Considering the dual role of iron in inflammation, we speculate the existence of a complex Pyk2-iron-inflammation signal axis. 

The study is limited by the use a small number of clinical samples obtained from single research center, which may be one of the factors underlying the inconsistency between clinical data and experimental results. Additional cohort studies are needed to corroborate the aforementioned findings to facilitate the development of therapeutic strategies targeting Pyk2.

## Conclusion

A Pyk2 inhibitor of ferroptosis is a promising therapeutic candidate against sepsis-related ALI. 

## Authors’ Contributions

All authors, J W, P B and Y L contributed equally to the design of the study, data collection and analysis, and the writing of the manuscript. All authors read and approved the final manuscript.

## Ethics approval and consent to participate

The study has been approved by the ethics committee of Beijing Chaoyang Hospital (No. 2022-ke-297). All the experiments were carried out according to principles of Helsinki Declaration. Informed consent was waived. All authors declare that they have consented for publication.
